# Urinary Exosomal miRNAs as biomarkers of bladder Cancer and experimental verification of mechanism of miR-93-5p in bladder Cancer

**DOI:** 10.1186/s12885-021-08926-x

**Published:** 2021-12-03

**Authors:** Hao Lin, Xiaojun Shi, Haoran Li, Jialiang Hui, Ruiyu Liu, Zihao Chen, Yuwen Lu, Wanlong Tan

**Affiliations:** 1 Department of Urology, Nanfang Hospital, Southern Medical University, Guangzhou, 510515 China; 2 Department of Radiation Oncology, Nanfang Hospital, Southern Medical University, Guangzhou, 510515 China

**Keywords:** Bladder cancer, Urinary exosomes, microRNA, Biomarker, BTG2

## Abstract

**Background:**

Bladder cancer (BC) is one of the most common malignancies globally. Early diagnosis of it can significantly improve patients’ survival and quality of life. Urinary exosomes (UEs)-derived miRNAs might be a promising biomarker for BC detection.

**Method:**

A total of 12 patients with BC and 4 non-cancerous participants (as healthy control) were recruited from a single center between March 2018 and December 2019 as the discovery set. Midstream urine samples from each participants were collected and high-throughput sequencing and differentially expression analysis were conducted. Combined with miRNA expression profile of BC tissue from The Cancer Genome Atlas (TCGA), miRNAs biomarkers for BC were determined. Candidate miRNAs as biomarkers were selected followed by verification with a quantitative reverse-transcription polymerase chain reaction assay in an independent validation cohort consisting of 53 BC patients and 51 healthy controls. The receiver-operating characteristic (ROC) curve was established to evaluate the diagnostic performance of UE-derived miRNAs. The possible mechanism of miRNAs were revealed by bioinformatic analysis and explored in vitro experiments.

**Results:**

We identified that miR-93-5p, miR-516a-5p were simultaneously significantly increased both in UEs from BC compared with healthy control and BC tissue compared with normal tissue, which were verified by RT-qPCR in the validation cohort. Subsequently, the performance to discover BC of the miR-93-5p, miR-516a-5p was further verified with an area under ROC curve (AUC) of 0.838 and 0.790, respectively, which was significantly higher than that of urine cytology (AUC = 0.630). Moreover, miR-93-5p was significantly increased in muscle-invasive BC compared with non-muscle-invasive BC with an AUC of 0.769. Bioinformatic analysis revealed that B-cell translocation gene 2(BTG2) gene may be the hub target gene of miR-93-5p. In vitro experiments verified that miR-93-5p suppressed BTG2 expression and promoted BC cells proliferation, invasion and migration.

**Conclusion:**

Urine derived exosomes have a distinct miRNA profile in BC patients, and urinary exosomal miRNAs could be used as a promising non-invasive tool to detect BC. In vitro experiments suggested that miR-93-5p overexpression may contribute to BC progression via suppressing BTG2 expression.

**Supplementary Information:**

The online version contains supplementary material available at 10.1186/s12885-021-08926-x.

## Background

Bladder cancer (BC) is the most common malignancy of the urinary tract worldwide [1]. About 75% of patients are classified as non-muscle-invasive BC (NMIBC), while the rest are muscle-invasive BC (MIBC) [2]. NMIBC features a high recurrence rate and MIBC patients usually have a poor prognosis because of recurrence or metastasis [2, 3]. Cystectomy is the main treatment of MIBC and has a 5–15% probability of pelvic recurrence that most often occurs within 6–18 months after surgery. Pelvic recurrences usually carry a poor prognosis, despite treatment, with median survival from 4 to 8 months [4]. The poor prognosis of BC is partially due to lack of an effective non-invasive means for early screening. At present, cystoscopy and pathological biopsy are the golden standard for diagnosing BC [5]. However, the invasiveness of cystoscopy limits its application as a regular screening tool for BC. Currently urine cytology is the most commonly used non-invasive diagnosis tool for BC, but is limited by low sensitivity [6]. Therefore, exploration of effective non-invasive biomarkers for screening BC can play a pivotal role in improving the prognosis and quality of lives of BC patients.

MicroRNAs (miRNAs) are endogenous 21-23 nt small non-coding RNAs with a length of approximately 21–23 nucleotides, which are capable of suppressing gene expression through post-transcriptional regulation. miRNAs are involved in various physiological and pathological procedures [7], which includes but not limit to, embryo development [8], oncogenesis [9, 10], and immune regulation [11]. miRNAs have been isolated and identified in many kinds of biofluids, such as plasma, serum, and urine [12], suggesting the potential of miRNAs as minimally invasive biomarkers of cancer [13]. But the instability of free miRNAs in biofluids hinders its application in clinical practice.

Exosomes are described as small membrane vesicles with a diameter of approximately 30–150 nm derived from cell endosomes [14]. They can be secreted into nearly all body fluids, including blood and urine, by nearly all kinds of cells [15]. The membrane construction contained a selection of miRNAs, mRNAs, lncRNAs, proteins, and lipids [16, 17]. Exosomes can act as messengers in cell-to-cell communication by transferring contained molecules and play important roles in tumorigenesis, progression and metastasis [18].

There is increasing evidence suggested the potential role for exosomal miRNA in early diagnosis of many diseases [19, 20]. However, systematic screening of urinary exosomal miRNA serving as BC biomarkers has not yet been studied. The purpose of this study is to identify urinary exosomes derived miRNA biomarkers as a non-invasive method to discriminate BC and clarify the mechanism of miR-93-5p in BC cells.

## Materials and methods

### Patients enrollment and sample collection

A total of 12 patients with BC (six NMIBC and six MIBC) and 4 non-cancerous participants (as healthy control) were recruited from Nanfang Hospital of Southern Medical University (Guangzhou, China) between March 2018 and December 2019 as the discovery set for discovering miRNAs suitable for biomarkers. An additional 104 participants (53 BC and 51 healthy control) were enrolled for validation. Written informed consent was obtained from each participant. This study was approved by the ethics committee of Nanfang Hospital. Midstream urine samples from each BC patients and healthy controls with 50 mL were collected, complying with the following criteria: urine samples from BC patients were collected before any antitumor therapies, such as surgery, chemotherapy or radiotherapy; urine samples of healthy controls were acquired from people who went through a medical check-up and showed no disease; and all these participants, showed no evidence of disease in other organs. BC was diagnosed based on histopathological findings. The tumor stage and grade were decided complied with the tumor-node metastasis (TNM) staging system and the WHO 2004 grading scheme, respectively [21]. Urine samples were immediately centrifuged at 3000 g for 20 min at 4 °C to remove cell debris, and the supernatant fluids were then collected and stored at − 80 °C until exosome extraction.

### Urinary exosome isolation

We conducted differential ultracentrifugation to isolate exosomes according to the standard method with a slight modification as previously depicted [22]. The aforementioned cell-free supernatant was centrifuged at 17,000 g for 30 min. Subsequently, supernatant was obtained and filtered with 0.22-μm filters (Millipore, Burlington, MA, USA) to remove microvesicles (200–1000 nm in diameter) and apoptotic bodies (800–5000 nm in diameter). Eventually, the supernatant that originated from the previous procedure was centrifuged at 110,000 g for 70 min at 4 °C in an ultracentrifuge (Beckman Coulter, Miami, FL, USA) to pellet exosomes from urine. After the supernatant was extracted, the exosomal pellet was suspended with 200 μL of phosphate-buffered saline (PBS) again.

### Transmission electron microscopy (TEM)

We used TEM to observed and identified the structure of isolated exosomes as previously reported and manufacturer’s protocols [23]. A total of 20 μL exosomes-enriched solution was placed on a copper mesh and incubated at room temperature for 10 min. After washing with sterile distilled water, the exosomes was contrasted by uranyloxalate solution for 1 min. The sample was then dried for 2 min under incandescent light. The copper mesh was observed and photographed under a JEM 1400 transmission electron microscope (JEOL, Tokyo, Japan).

### Nanoparticle tracking analysis (NTA)

Exosomes isolated from urine were processed for nanoparticle tracking analysis (NTA) with NanoSight NS300 instrument (Malvern, UK). Briefly, exosomes were diluted in 1 mL PBS and mixed well, and then the diluted exosomes were injected into the NanoSight NS300 instrument (Malvern, UK). Instrument settings were set according to the manufacturer’s software manual. Particles were automatically tracked and sized based on the in-build NanoSight Software NTA 3.1 Build 3.1.46. Filtered PBS was used as a control.

### Western blot analysis

Total protein was extracted in RIPA lysis buffer (89,900, Thermo Fisher Scientific, Waltham, MA, USA). Equal loading of extracted protein was denatured in 5× sodium dodecyl sulfonate (SDS) buffer and subjected to western blot analysis (10% SDS-polyacrylamide gel electrophoresis; 50 μg protein/lane) using rabbit polyclonal antibody CD63 (abs-134,386, Absin, Shanghai, China), TSG101(abs-122,785, Absin, Shanghai, China), BTG2(ab-24,460, Abcam, UK), Calnexin (ab-133,615, Abcam, UK) and GAPDH (abs-133,958, Absin, Shanghai, China). The proteins of interest were detected on a gel imaging system using ECL western blotting substrate (Thermo Fisher Scientific, Waltham, MA, USA) and band density was analysed with ImageJ software.

### RNA extraction and qRT-PCR

Total RNA extraction from exosomes, tissues and cultured cells was performed with the Trizol Reagent (15,596,026, Invitrogen, Carlsbad, CA, USA) as previously reported [24]. The resulting RNA pellet was stored at − 80 °C until further analysis. RNA was quantified and assessed by NanoDrop® ND-2000 (Thermo Fisher Scientific, Waltham, MA, USA). The expression of three mature miRNAs namely hsa-miR-93-5p, hsa-miR-516a-5p, and hsa-miR-940 (Table I) was quantified using TaqMan single® microRNA assays (442,975, Applied Biosystems®) in accordance with the manufacturer’s protocol. The same amount of *Caenorhabditis elegans* cel-39 miRNA was spiked into each exosomes sample as an external calibration for RNA extraction, reverse transcription, and miRNA amplification. Real-time PCR was performed with ABI 7300 Real-Time PCR System (Applied Biosystems, Foster City, CA, USA). Relative gene expression was calculated using the 2^-□□Ct^ method [25] and normalized to spike-in control cel-miR-39 or endogenous control U6 snRNA.

### Library preparation and sequencing

High throughput sequencing technology for exosomal miRNAs from urine was performed as manufacturer’s recommendations and previously reported [23, 26]. For small RNA libraries, a total amount of 2.5 ng RNA per sample was used as input material for the RNA sample preparations. Sequencing libraries were generated using NEB Next Multiplex Small RNA Library Prep Set for Illumina (NEB, Ipswich, MA, USA) following the manufacturer’s recommendations. Index codes were added to attribute sequences to each sample. Adapter Ligated RNA was mixed with ProtoScript II Reverse Transcriptase, Murine RNase Inhibitor, First Strand Synthesis Reaction Buffer (NEB, Ipswich, MA, USA) and incubated for 60 min at 50 °C. We mixed the purified PCR product (25 μL) with 5 μL of Gel Loading Dye, loaded 5 μL of Quick-Load pBR322 DNA-MspI Digest in on the 6% PAGE 10-well gel, run the gel for 1 h at 120 V. For miRNA, the bands corresponding to ~ 150 bp were isolated. At last, the library quality was assessed with both the 2100 Bioanalyzer System (Agilent, Santa Clara, CA, USA) and qPCR. After cluster generation, the libraries were sequenced on an Illumina Hiseq X ten platform and 150 bp paired-end reads were generated.

### Differential expression analysis of miRNA

Differential expression analysis of exosomal miRNAs was performed as previously reported [23]. With the help of software Bowtie, clean reads were aligned and compared with sequences in databases including Silva, GtRNAdb, Rfam, and Repbase respectively. Reads with more than 10%N, low quality, length > 32 nt/< 16 nt, or trimming 3′ adapter from the end of reads (no mismatch) were filtered. After filtering unwanted sequences, such as ribosomal RNA (rRNA), transfer RNA (tRNA), small nuclear RNA (snRNA), and small nucleolar RNA (snoRNA), remaining reads were compared with miRNAs from miRbase and Human Genome (GRCh38) to identify known miRNAs as well as the prediction of new miRNAs. Reads counts were generated according to the mapping results of miRDeep2, which was used to calculate TPM. Level 3-normalized miRNA expression data for 410 bladder cancer patients were obtained from TCGA (https://portal.gdc.cancer.gov/) by using R language package TCGAbiolinks. Datasets from 19 solid normal tissue samples of bladder urothelial as non-cancerous control set were also obtained. Then we used the TMM method [27] in edgeR to normalize the TPM of miRNA. A miRNA was regarded as differentially expressed if it exhibited |log2(Fold Change)| > 1(*p* < 0.05).

### Target gene prediction, GO/KEGG pathway enrichment analysis and PPI network

Potential target genes of selected miRNA were predicted by miRWALK2.0, an online archive of data on miRNA-target interactions [28] for further analysis. In total, 12 servers with miRWalk, miRMap, MicroT4, miRNAMap, TargetScan, PICTAR2, miRBridge, PITA, miRanda, RNAhybrid, miRDB, RNA22 were used. Only those genes projected by more than six of the servers were recognized as target genes. Since miRNAs could down-regulated the expression of target genes, the low-expressed genes in bladder cancer were acquired through bioinformatic analysis from public data from TCGA database. The overlapping genes among the down-regulated genes in BC and the predicted target genes, were viewed as promising targets of selected miRNA in BC. The Gene Ontology (GO) analysis, which include biological processes (BPs), cellular components (CCs), and molecular functions (MFs), were conducted by clusterProfiler R package. The functional annotation of the underlying target genes was then elucidated by Kyoto Encyclopedia of Genes and Genomes (KEGG) pathway analysis with clusterProfiler R package. In addition, a Protein-Protein Interaction (PPI) network was constructed to reveal the hub genes of the potential target genes on STRING, a web portal for undermining the integrated function of multiple genes [29].

### Cell lines and cell culture

The human BC cell lines T24, UM-UC-3, as well as one normal bladder cell line SV-HUC-1, were purchased from the Shanghai Institute of Cell Biology, Shanghai, China. These cell lines were cultured according to manufacturer’s recommendations and previously reported. Cell lines were maintained in Roswell Park Memorial Institute 1640 medium (RPMI1640; Gibco, Carlsbad, CA, USA) with 10% fetal bovine serum (FBS; Biological Industries, Cromwell, CT, USA), under a humidified atmosphere of 5% CO_2_ at 37 °C. The cell culture medium was changed every 2–3 days, and the cells were passaged with 0.25% trypsin-EDTA (Gibco, Carlsbad, CA, USA) and grown to 90% confluence.

### Reagents and transfection

The hsa-miR-93-5p mimic (miR-93-5p mimic; miRBase accession MIMAT0000093; sense: 5′-CAAAGUGCUGUUCGUGCAGGUAG-3′), the negative control (NC) of the mimic duplex (NC, sense:5′-UUCUCCGAACGUGUCACGUTT-3′), the hsa-miR-93-5p inhibitor (miR-93-5p inhibitor; sense: 5′-CACUUAUCAGGUUGUAUUAUAA-3′) and the negative control duplex of the inhibitor (inhibitor NC, sense:5′-CAGUACUUUUGUGUAGUACAA-3′) with no significant homology to any known human sequences were used for gain-of-function studies. The RNA duplexes were chemically synthesized by GenePharma, Shanghai, China. Oligonucleotide transfection was performed using Lipofectamine 2000 reagents (11,668,019, Invitrogen, Carlsbad, CA, USA) in accordance with the manufacturer’s protocol.

### Dual-luciferase reporter assay

Dual-luciferase reporter assay was performed as previously reported [30]. Olgonucletide pairs that contained the desired miR-93-5p target region or mutant target region were designed and ordered from GenePharma, Shanghai, China. After annealing, these double-stranded segements were inserted into pmirGLO Dual-Luciferase miRNA Target Expression Vector (Promega, Madison, WI, USA), between the SacI and SalI sites. The insertions were verified by sequencing. Dual-luciferase assays were performed using 1 × 10^4^ T24 cells per well in a 96-well plate (Corning, Acton, MA, USA). After the cells attached for 8 h, they were cotransfected with 50 ng of miRNA mimics or control miRNA. After 48 h, a Reporter Assay System Kit Pierce™ (16,186, Thermo Fisher Scientific, Waltham, MA, USA) was used to measure the luciferase activity. There were three replicates for each transfectant. Firely luciferase activity was normalized to constitutive Renilla luciferase activity.

### Cell growth/cell viability assay (cell count Kit-8 assay)

Cell Count Kit-8 assay was performed to evaluate the cell viability as previously reported [30, 31].T24 or UM-UC-3 cells were plated in 96-well plates with ~ 4 × 103 cells per well. After overnight incubation, the cells were transfected with the RNA duplex (miR-93-5p mimic,miR-93-5p inhibitor, or NC) for 2–3 days with concentrations 50 nM. At different time points, the medium was removed and WST-8 (Dojindo Laboratories, Kumamoto, Japan) was added to each well. After the 96-well plate was incubated at 37 °C for 1 h, the absorbance of the solution was measured spectrophotometrically at 450 nm with an MRX II absorbance reader (Dynex Technologies, Chantilly, VA, USA).

### Cell migration and invasion assay

The cell migration and invasion assay were performed according to the standard method with a slight modifications as previously reported [30, 31]. For the invasion assay, the inserts were coated with Matrigel (BD Bioscience, Franklin Lakes, NJ, USA) on the upper surface. After transfection, 8 × 104 cells were suspended in 0.2 ml serum-free medium and added to the inserts. Then, 0.6 ml RPMI-1640 medium with 10% FBS was added to the lower compartment as a chemoattractant. After incubation at 37 °C for 24 h, the cells on the upper surface of the membrane were carefully removed using a cotton swab and cells on the lower surface were fixed with 100% methanol and stained with 0.1% crystal violet. Five visual fields of 200× magnification of each insert were randomly selected and counted under a light microscope (Olympus, Japan).

### Statistical analysis

Statistical tests were performed using R 3.5.1 (www.r-project.org). Data are presented as median (interquartile interval). All tests were two-tailed and False Discovery Rate (FDR) was controlled for multiple comparisons. The differences in the expressions of UE-derived miRNAs between BC patients and healthy controls were assessed by non-parametric Mann–Whitney U test. Diagnostic accuracy of candidate miRNAs or their combinations was assessed by receiver operating characteristic (ROC) curves analysis. Correlation analysis was conducted by Pearson’s correlation method. Survival curves were generated by Kaplan–Meier method, and the difference was compared by log-rank test. Packages plyr and reshape2 were used for data sorting and restructuring. VennDiagram, pheatmap, and ggplot2 were used for visualization of results. A *P* value < 0.05 was considered as statistically significant.

## Results

### Characterization of urinary exosomes

**Fig. 1:**
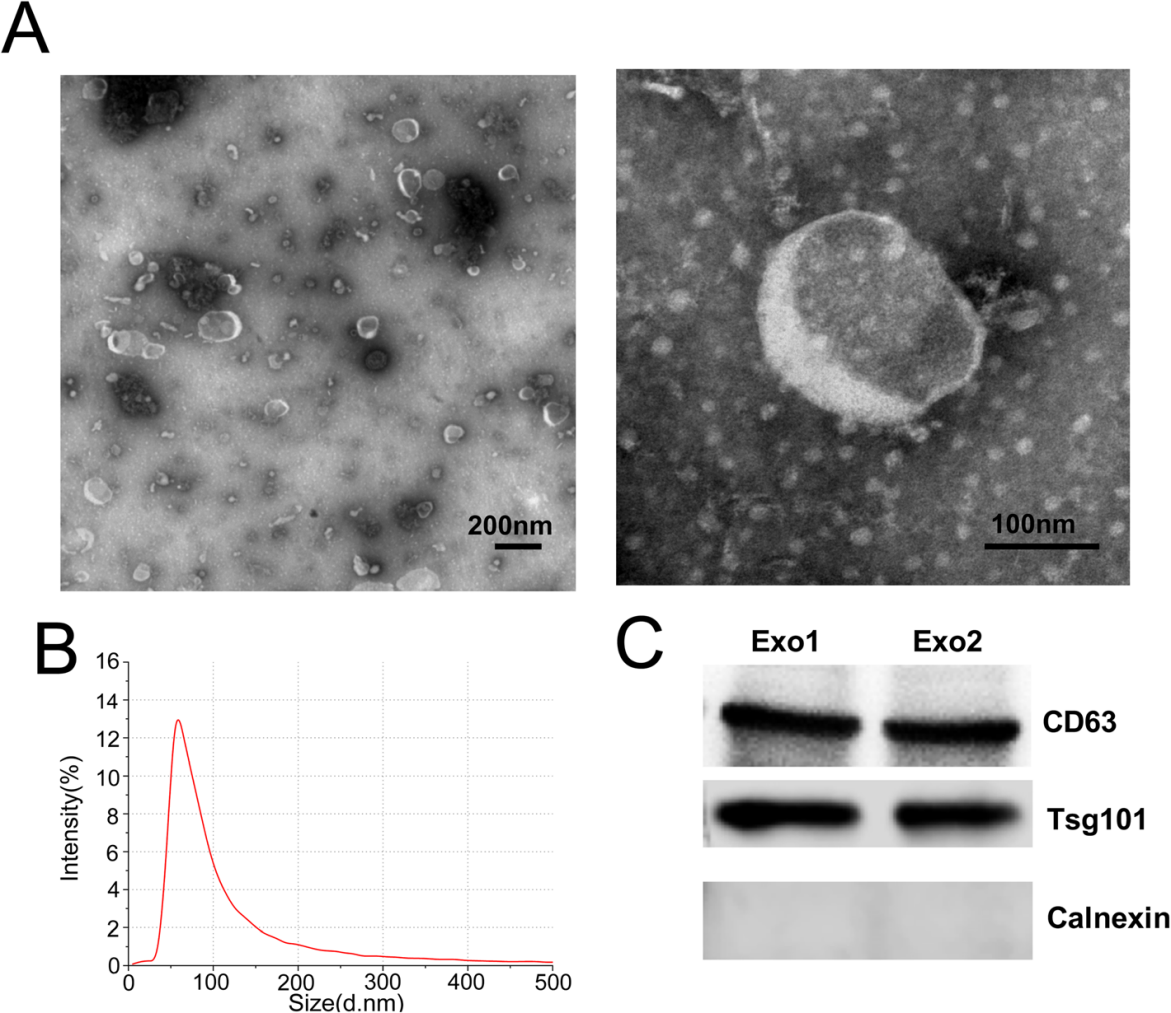
Characterization of urinary exosomes. (A) TEM images showed that exosomes were oval or bowl-shaped capsules without the nucleus. (B) NTA results suggested that UEs enriched from urine were about 50 nm–200 nm in diameter. (C) Exosomes markers CD9, CD63 and TSG101 were detected in UEs, and Calnexin, a negative marker of exosomes was absent in isolated UEs samples. Exo, exosomes

The urinary exosomes collected from participants were characterized using TEM, NTA and western blotting. TEM and NTA analysis showed that UEs have a diameter of 50–200 nm with a cup-shaped membrane (Fig. 1A,B). Western blotting of UEs demonstrated the presence of CD63, and TSG101,which are exosome markers (Fig. 1C). On the contrary, Calnexin, a negative marker of exosome was absent (Fig. 1C). Collectively, these data indicated that exosomes existed in urine, which laid a foundation for further study of exosomal biomarkers.

### Urine exosome-derived miRNAs profile analysed by high throughput sequencing

To identify a global differential expression profile of the exosomes derived miRNA from the urine of BC patients, testing set (6 MIBC patients, 6 NMIBC, 4 controls) were recruited for miRNA sequencing. The basic information of the discovery set was shown in Table 1. Fifty-one miRNAs were found up-regulated, and 22 down-regulated in urinary exosomes from BC patients compared with healthy control group. Compared to NMIBC patients, 40 miRNAs were found up-regulated, and 21 down-regulated in urinary exosomes from MIBC patients. Heatmaps showed the markedly different profiles of urinary exosomal miRNAs (Supplement Fig. 1A, B). DEMs analysis was also conducted in TCGA dataset, 106 miRNAs were found up-regulated, and 26 down-regulated in BC tissue than adjacent normal tissue. Intersection analysis and Venn diagram showed there were 3 DEMs shared between those three analyses, among which were all up-regulated (Supplement Fig. 1C, D). The three DEMs, miR-93-5p,miR-516a-5p and miR-940 were selected for further validation and analysis.

**Table 1:** Basic clinical information of patients with bladder cancer and health control for sequencing

ID	Gender	Age	T stage	Pathological Grade
MIBC1	male	62	T2	High
MIBC2	male	65	T2	High
MIBC3	male	61	T3	High
MIBC4	female	59	T2	High
MIBC5	female	67	T2	High
MIBC6	female	63	T3	High
NMIBC1	male	68	T1	Low
NMIBC2	male	58	T1	Low
NMIBC3	male	61	Ta	Low
NMIBC4	female	65	T1	High
NMIBC5	female	62	T1	High
NMIBC6	female	62	Ta	Low
Control1	male	60	/	/
Control2	male	58	/	/
Control3	female	62	/	/
Control4	female	63	/	/

### Validation results of urinary exosomal miRNAs by RT-qPCR

**Fig. 2:**
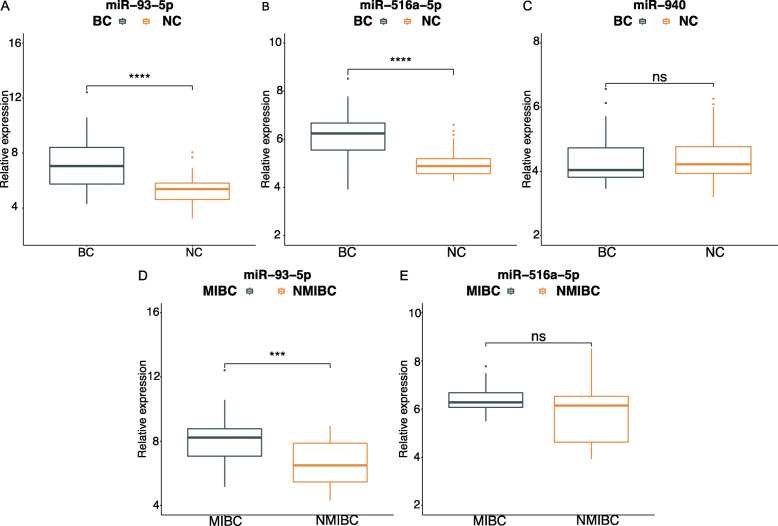
Validation analysis of selected miRNAs in the validation set. Relative expression level of UE-derived (A) miR-93-5p, (B) miR-516a-5p and (C)miR-940 between BC patients and healthy control. Relative expression level of UE-derived (D) miR-93-5p and (E) miR-516a-5p between MIBC patients and NMIBC. Relative expression was calculated using the 2-횫횫Ct method and normalized to spike-in control cel-miR-39. *** represents p<0.001, **** represents p<0.0001, ns represents not significant

We then performed RT-qPCR assays to confirm the results of the sequencing in the validation set (53 BC and 51 healthy control). Basic information of the validation set was shown in Table 2. No significant distinction was present in the age and gender composition between the healthy control group (male/female: 40/13; median age (IQR): 65 (52–69)) and the healthy control group (male/female: 35/16; median age (IQR): 62 (54–68)). In accordance with the results of the sequencing, miR-93-5p and miR-516a-5p were significantly up-regulated in BC patients compared with the healthy controls (Fig. 2A, B). There were no significant differences in expression level of urinary exosomal miR-940 between BC and healthy control (Fig. 2C). The expression level of miR-93-5p and miR-516a-5p were also compared between patient groups with different stage, including MIBC and NMIBC. MiR-93-5p was verified to be significantly up-regulated in MIBC patients compared with NMIBC (Fig. 2D). However, there were no significant differences in expression level of miR-516a-5p between the subtypes (Fig. 2E).

**Table 2:** Clinical features of patients with BC and health control for verification

Variables	BC	Healthy control	***P***-value
	*n* = 53	*n* = 51	
**Median age,(IQR)**	65 (52–69)	62 (54–68)	0.59^a^
**Gender**			0.504^b^
Male	40	35	
Female	13	16	
**pT stage**			/
Ta-T1	32	/	
T2-T4	21	/	
**Grade**			/
Low	22	/	
High	31	/	
**Lymph node**			/
Positive	6	/	
Negative	47	/	

^a^Mann-Whiteney U-test; ^b^Two-side test

### ROC curve analysis

**Fig. 3:**
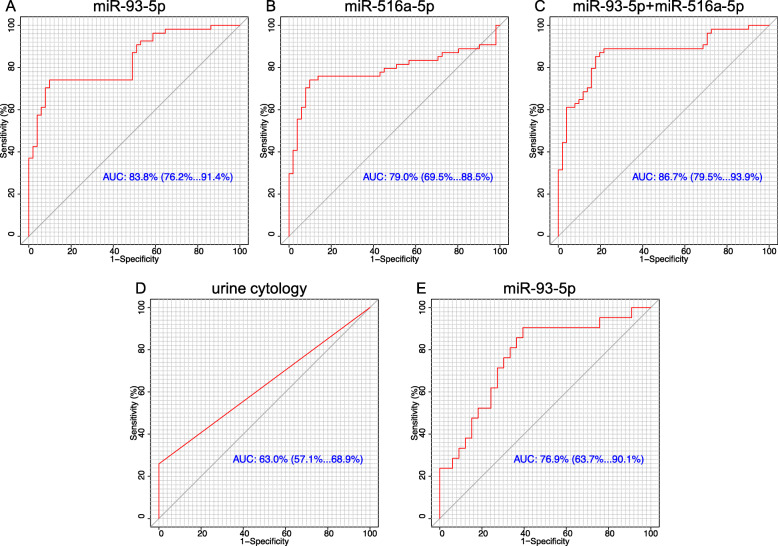
Diagnostic performance of miR-93-5p,miR-516a-5p and urine cytology as biomarkers of BC. ROC curve analysis showing the diagnostic performance for BC detection of UE-derived (A) miR-93-5p,(B) miR-516a-5p,(C) miR-93-5p plus miR-516a-5p and(D) urine cytology. Diagnostic performance for distinguishing from MIBC and NMIBC of urinary exosomes-derived (D)miR-93-5p

To evaluate the potential diagnostic value of identified miRNA, ROC analysis was performed and AUC was calculated in the validation set. The AUC value of miR-93-5p and miR-516a-5p for detecting BC was 0.838 (95% CI: 0.762–0.914) and 0.790 (95%CI: 0.695–0.885), respectively (Fig. 3A,B). The corresponding sensitivity and specificity were 74.1 and 90.2%,72.9 and 89.9%. There were no significant difference in AUC between miR-93-5p and miR-516a-5p (p>0.05). Considering that combinations of tumor markers can improve the diagnostic accuracy, logistic regression was performed to combine the miR-93-5p and miR-516a-5p. The AUC of the combination panel was 0.867 (95% CI: 0.795–0.939), with the sensitivity and specificity values of 85.2 and 82.4%, respectively (Fig. 3C). The combination of the two miRNA showed no significant difference compared with single miRNA(p>0.05). Currently, urine cytology is widely used in clinical practice, but it has relatively poor sensitivity. Therefore, we compared the diagnostic performance between the panel and urine cytology. As expected, the AUC of urine cytology for BC detection was 0.630 (95% CI =0.571–0.689, sensitivity = 25.9% and specificity = 100%) (Fig. 3D), which was significantly lower than that of the miRNA. In the patients group, ROC analysis was performed to evaluate the value of miR-93-5p in distinguishing the MIBC and NMIBC. The AUC was 0.769 (95% CI =0.637–0.901, sensitivity = 90.5% and specificity = 60.6%) (Fig. 3E).

### Correlation between 2 UE-derived miRNAs and clinicopathological characteristics

Next, we analyzed the correlation between the 2 UE-derived miRNAs and clinicopathological characteristics of the BC patients. Results demonstrated overexpression of UE-derived miR-93-5p were correlated with advanced T stage (r = 0.43, 95%CI:0.17–0.63, *p* < 0.05), which was accordance with the RT-qPCR results. However, we did not find any significant association between the 2 miRNAs and age, sex or tumor grade (all at *p* > 0.05) (Table 3).

**Table 3:** Correlation between miRNA expression and BC clinical information

Clinical character	n	miR-93-5p	p-value	miR-516a-5p	p-value
**Age (year)**			0.84		0.49
≤ 64	22	6.95 (6.35–8.40)		7.15 (6.63–7.78)	
>64	31	6.98 (5.35–8.31)		6.98 (5.71–7.58)	
**Gender**			0.85		0.68
Male	40	6.96 (6.07–8.38)		7.04 (6.15–7.58)	
Female	13	6.79 (5.58–8.13)		7.07 (5.89–7.74)	
**pT stage**			<0.01		0.06
Ta-T1	32	6.39 (5.35–7.85)		6.98 (5.41–7.64)	
T2-T4	21	8.06 (6.96–8.63))		7.14 (6.84–8.22)	
**Grade**			0.15		0.08
Low	22	6.39 (5.36–8.17)		6.87 (5.54–7.69)	
High	31	7.24 (6.74–8.46)		7.08 (6.82–7.70)	
**Lymph node**			0.47		0.77
Negative	47	6.96 (5.60–8.39)		7.03 (5.86–7.65)	
Positive	6	6.63 (6.05–7.28)		7.27 (6.39–7.82)	

### Promising target genes collection

From miRWALK2.0, 2140 genes targeted by miR-93-5p in BC predicted by more than six algorithms were obtained. A total of 1902 down-expressed genes in BLCA were collected on the basis of TCGA. After intersection, a total of 289 predicted target genes of miR-93-5p were collected (Supplement Fig. 2).

### GO/KEGG analysis PPI network

Bioinformatic analysis including GO enrichment, KEGG pathway and PPI network were conducted to reveal potential function of the promising target genes in BC patients. The results of GO enrichment and KEGG pathway analysis showed that target genes of miR-93-5p were mainly enriched in RNA polymerase II-specific DNA-binding transcription activator activity, actin binding and PI3K-Akt signaling (Supplement Fig. 3). PPI network were conducted using the public database STRING to revealed the protein-protein interaction of target genes in bladder cancer (Supplement Fig. 4). We used clustering coefficent, a parameter that demonstrated the degree to which nodes in a net tend to cluster to predict the hub genes and genes of the PPI network [32]. The genes of which rank top 5 were selected as hub genes. The PPI analysis revealed NFIC, TIMP2, BTG2, SNX9 and PKD1 as hub target genes of miR-93-5p.

### Correlation between the hub genes and BC prognosis

**Fig. 4:**
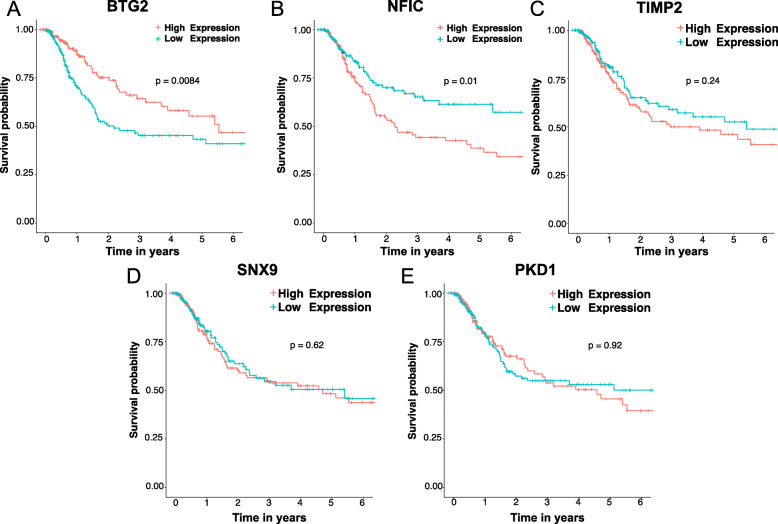
Kaplan-Meier survival curves of hub genes expression based on TCGA BC cohort

To explore the relationship between prognosis of BC patients and hub target genes expression of miR-93-5p, Kaplan-Meier survival analysis was performed according to TCGA dataset. The results showed that BC patients with lower levels of BTG2, the target hub gene of miR-93-5p, might have worse outcomes(p<0.01, Fig. 4A). However, none of the other hub genes expression level were correlated with the overall survival of BC patients (all at *p* > 0.01, Fig. 4B, C, D, E). According to the survival analysis, expression level of BTG2 is related to the prognosis of BC patients. B-cell translocation gene 2 (BTG2), the first gene identified in the BTG/TOB gene family, is proved to be involved in many biological activities in cancer cells [33]. The BTG2 expression is downregulated in many human cancers acting as a tumor suppressor, including bladder cancer [34]. It is an instantaneous early response gene and plays important roles in cell differentiation, proliferation, DNA damage repair, and apoptosis in cancer cells [33–36]. According to the aforementioned results of bioinformatic analysis, we assumed that up-regulated miR-93-5p expression might play an oncogenic role in bladder cancer via targeting and suppressing BTG2 to cell proliferation, migration and invasion. We eventually decided to study the correlation between gene BTG2 and miR-93-5p.

### MiR-93-5p is up-regulated in BC tissues and cell lines

To evaluate the expression level of miR-93-5p in BC tissue, qRT-PCR was performed in 10 pairs of clinical BC tissues and adjacent non-cancerous tissues (the clinical characteristics of the patients are shown in Table 4). The expression level of miR-93-5p was frequently higher detected in tumor tissues than in non-tumor tissues (Supplement Fig. 5A, 8 out of 10 displayed a upregulation pattern). In two different urinary BC lines (T24 and UM-UC-3), miR-93-5p was also higher expressed in comparison with the non-tumor urothelial cell line SV-HUC-1 (Supplement Fig. 5B).

**Table 4:** Patient information

Patient ID	Gender	Age	TNM Stage	Pathological Grade
1	Male	77	T2bN2M0	High
2	Male	62	T2bN2M0	High
3	Female	56	T2aN0M0	Low
4	Male	76	T4N0M0	High
5	Male	69	T2bN2M0	High
6	Female	78	T3N0M0	High
7	Male	52	T2bN0M0	High
8	Male	56	T2aN0M0	High
9	Male	52	T2bN0M0	High
10	Male	62	T2bN2M0	High

### MiR-93-5p promotes BC cells proliferation

**Fig. 5:**
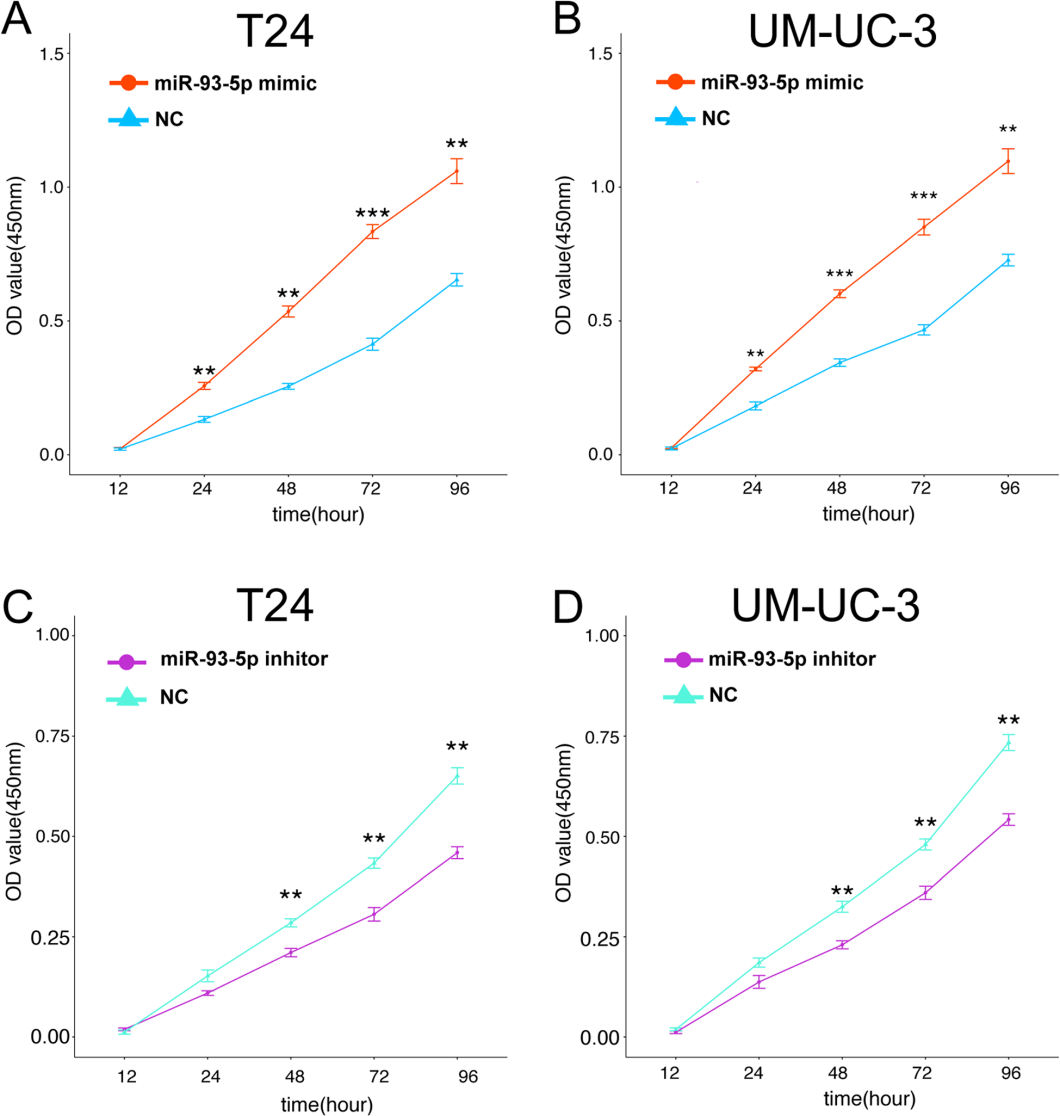
The impact of miR-93-5p level on proliferation of bladder cancer cells. (A) Cell counting kit-8(CCK-8) assay. BC cells were transfected with 50 nM miR-93-5p mimic or negative control (NC) for 12、24、48、72、96 h.The miR-93-5p mimic can promote the proliferation in BC cells (B) BC cells were transfected with 50 nM miR-93-5p inhibitor or negative control (NC) for 12、24、48、72、96 h.The miR-93-5p inhibitor can suppress the proliferation in BC cells. ** represents p<0.01, *** represents p<0.001

To assess the role of miR-93-5p in the regulation of BC proliferation in vitro, T24 and UM-UC-3 cells were transfected with miR-93-5p mimics, inhibitor, mimic negative control or inhibitor negative control. The change of expression level of miR-93-5p after transfection were detected using qRT-PCR (Supplement Fig. 5C, D). A CCK-8 assay was performed to detect the effect of miR-93-5p treatment on BC-cell proliferation, and showed that the both T24 and UM-UC-3 cell proliferation rates were significantly increased after miR-93-5p-mimic transfection, and conversely, was attenuated in response to miR-93-inhibitor transfection (Fig. 5). In a word, these findings indicate that miR-93-5p promotes proliferation capability of BC cell.

### MiR-93-5p promotes BC migration and invasion in vitro

**Fig. 6:**
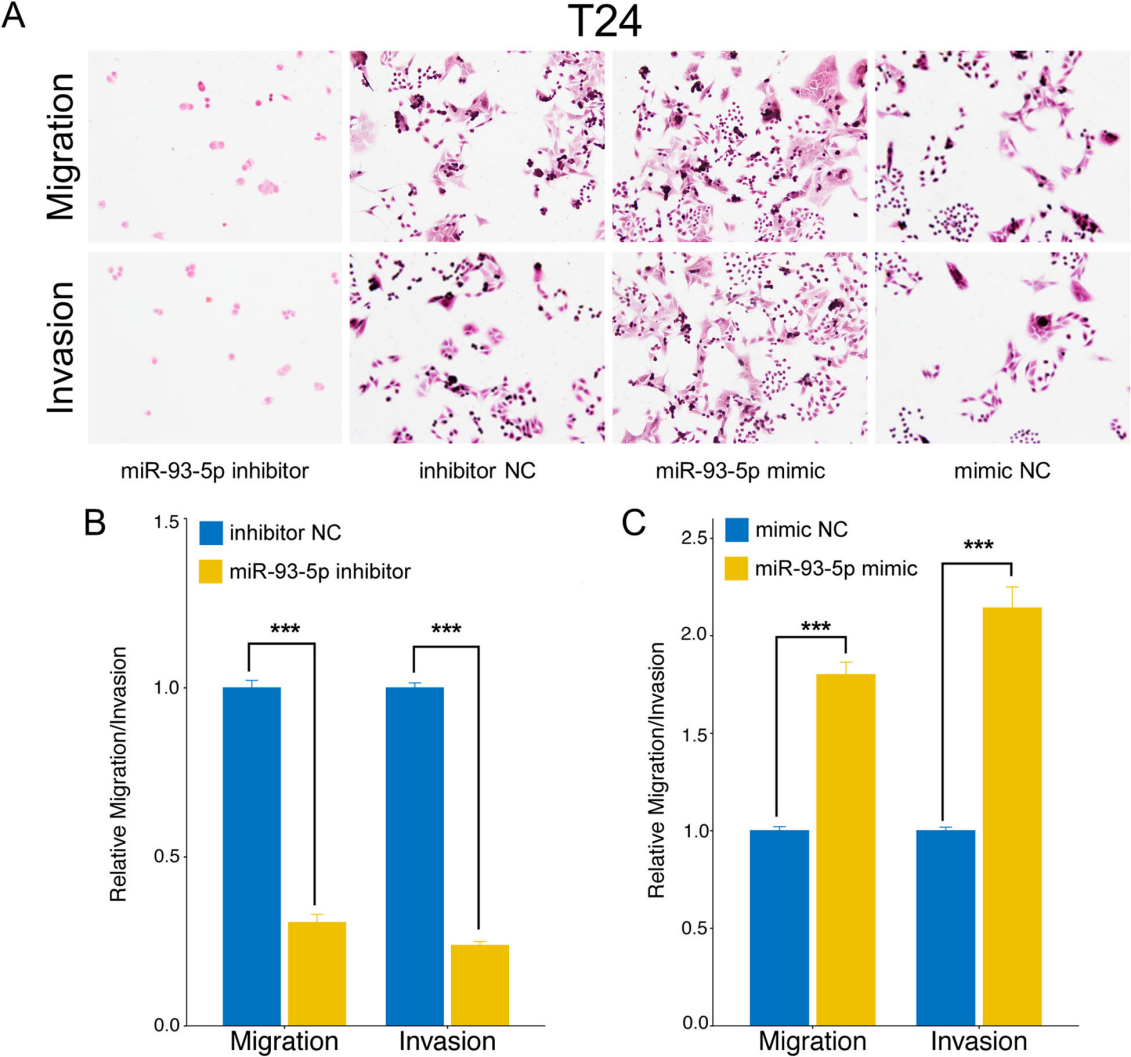
The impact of miR-93-5p level on migration and invasion of bladder cancer cell line T24. (A) Transwell analysis of T24 after transfection. (B) MiR-93-5p inhibitor suppresses migration and invasion ability of T24 cells compared with NC. (C) MiR-93-5p mimic promotes migration and invasion ability of T24 cells compared with NC. The relative levels were presented as the fold change referred to corresponding NC. *** represents p<0.001

**Fig. 7:**
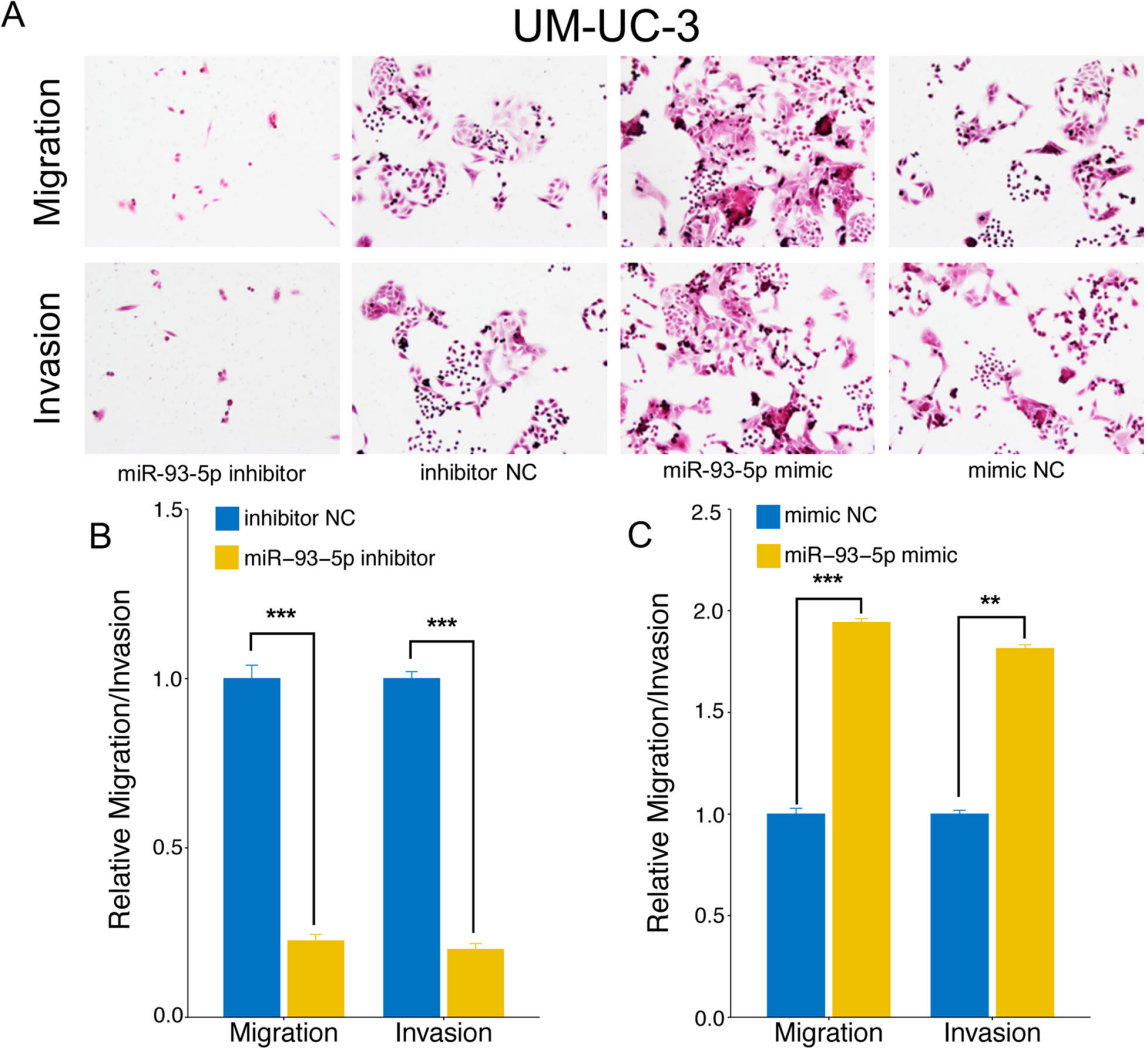
The impact of miR-93-5p level on migration and invasion of bladder cancer cell line UM-UC-3. (A) Transwell analysis of UM-UC-3 after transfection. (B) MiR-93-5p inhibitor suppresses migration and invasion ability of T24 cells compared with NC. (C) MiR-93-5p mimic promotes migration and invasion ability of T24 cells compared with NC. The relative levels were presented as the fold change referred to corresponding NC. **represents p<0.01; ***represents p<0.001

A transwell assay was conducted to investigate whether miR-93-5p treatment affected BC-cell migration and/or invasion, and revealed that miR-93-5p upregulation increased both the migration and invasion ability of T24 and UM-UC-3 cells. In contrast, miR-93-5p downregulation induced a significant reduction in both cell migration and invasion. All results were shown in Fig. 6 and Fig. 7.

### BTG2 is direct target gene of miR-93-5p

**Fig. 8:**
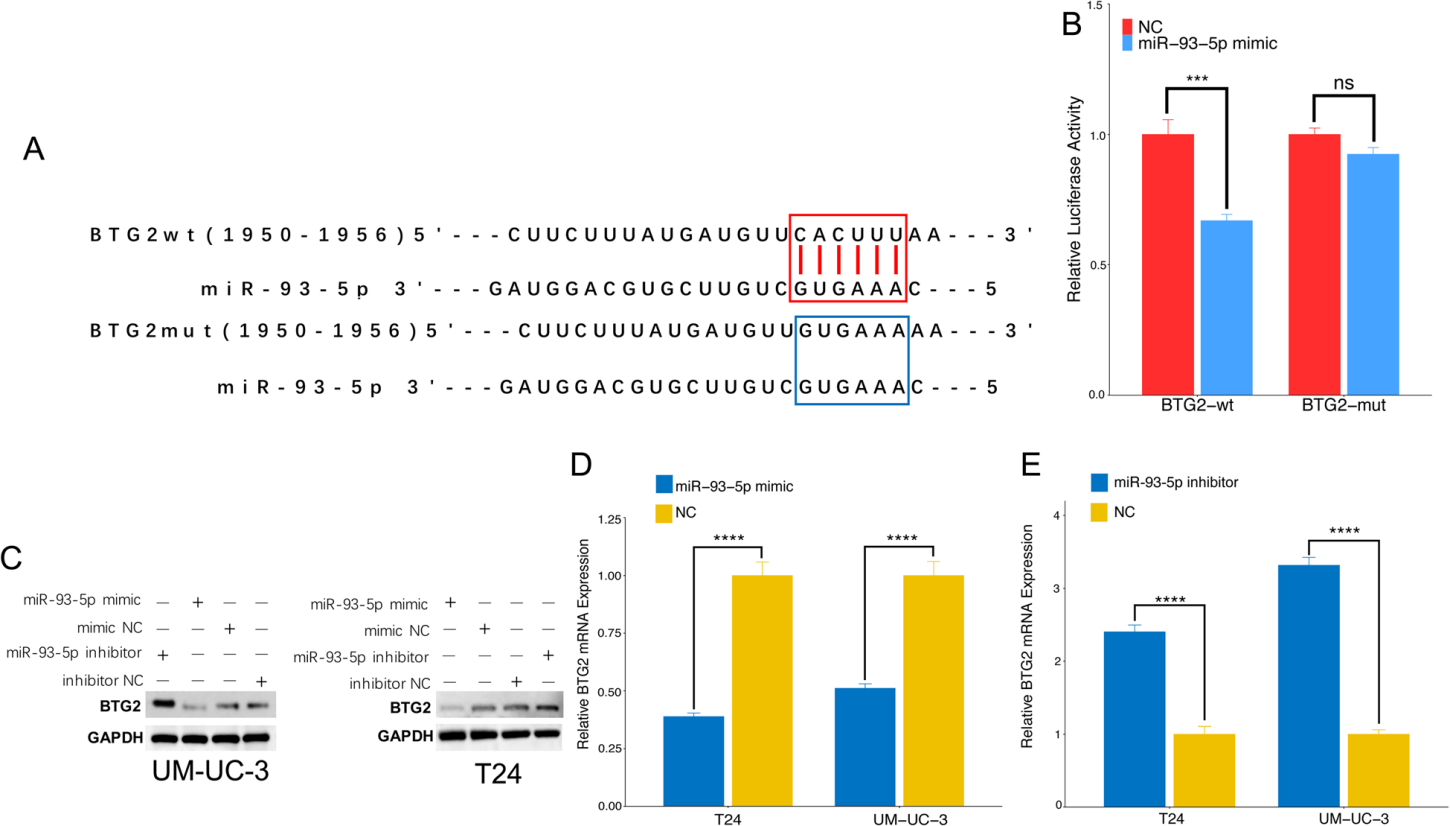
BTG2 is direct target of miR-93-5p. (A) Schematic representation of the miR-93-5p predicted binding sites in the 3′-UTRs of BTG2 mRNAs and 3′-UTR-mutated alignments. (B) Dual-luciferase reporter assay. The luciferase activities of the mutated vectors of BTG2 were unaffected by the transfection of miR-93-5p.(C) Western blotting assay. MiR-93-5p significantly inhibited the expression of BTG2. (D),(E). qRT-PCR analysis. MiR-93-5p significantly inhibited expression level of BTG2 mRNA in T24 and UM-UC-3 cells. The relative levels were presented as the fold change referred to corresponding NC. *** represents p<0.001, **** represents *p* < 0.0001, ns represents not-significant

We performed dual-luciferase report analysis to investigate whether BTG2 acts as miR-93-5p target. The Targetscan (http://www.targetscan.org/) database was used to designed the wildtype or mutated vectors (Fig. 8A). The results of luciferase report showed that miR-93-5p transfection significantly suppressed the luciferase activity induced by BTG2, conversely, the mutated vectors was not affected by miR-93-5p(Fig. 8B). The qRT-PCR and western blotting assay showed that the protein and mRNA level of BTG2 were significantly decreased and increased in response to miR-93-5p up- and downregulation, respectively.(Fig. 8C, D, E). Taken together, these in vitro experiments data verified the aforementioned bioinformatic analysis results which suggested that miR-93-5p can promote bladder cancer cells proliferation, migration and invasion abilities via inhibiting the target gene, BTG2.

## Discussion

Compared with other clinical samples from patients such as blood, urine has its unique advantages in clinical application: acquired non-invasively and easily accessible, which makes it a suitable source of biomarkers for many diseases [37]. Exosomes, extracellular microvesicles with diameter 30-150 nm, secreted by nearly all kinds of cells including cancer cells exist in biofluids, has been identified to contain bioactive molecule such as RNA (mRNA, miRNA, lncRNA), protein and lipid from original cells [38]. Moreover, the membrane structure of exosomes protect the contained molecule from degradation by enzymes [22]. Several studies have indicated that compared with cell-free urine, miRNAs were much more enriched and stable in urine-derived exosomes, and aberrant expression of particular miRNAs in exosomes might reflect the change in biological processes of disease [39]. Thus, urine-derived exosomal miRNA has attracted much attention from researchers as a new diagnostic tool to screen for disease [20, 40]. For instance, researchers had found that the urinary exosomes-derived miR-181a in patients with chronic kidney disease was significantly decreased compared with health control, making it a potential indicator for CKD diagnosis. As for bladder cancer, urine-derived exosomes had also been investigated as biomarkers. Zhan [41] and et.al developed a urinary exosome-derived lncRNA panel (MALAT1, PCAT-1 and SPRY4-IT1) for diagnosis and recurrence prediction of bladder cancer in a cohort consist of 368 urine samples. However, the sample the signature of exosomal miRNAs and its performance in diagnosis in urine for BC patients has not been sufficiently examined.

In the present study, a comprehensive analysis of urinary exosome-derived miRNA profile of BC patients and non-cancerous controls was performed with high throughput sequencing combined with RT-qPCR assays. We discovered that the expression profile of miRNAs in urinary exosomes from BC patients was markedly different from that of healthy controls, and identified 51 miRNAs up-regulated and 22 miRNAs down-regulated in BC samples compared to controls. In order to narrow the range of candidate miRNAs into miRNAs associated with development of bladder cancer to maximize the success rate of urinary exosomes biomarker verification, we identified the total 61 differentially expressed miRNAs (40 up-regulated and 21down-regulated) between MIBC and NMIBC. Then we selected miRNAs (106 up-regulated, 26 were down-regulated) that were also differentially expressed in the TCGA database as biomarker candidates. The reason why we compare our data side by side with differentially expressed miRNAs identified from TCGA is based on the assumption that miRNAs differentially expressed in bladder tumor is more likely to be a valid exosome-associated biomarker of BC. Finally, after the intersection, miR-93-5p, miR-516a-5p and miR-940(all up-regulated) were selected for further validation. By RT-qPCR assay validation arranged in an independent cohort, miR-93-5p and miR-516a-5p were validated to be significantly and steadily increased in BC patients. The ROC analysis showed that miR-93-5p and miR-516a-5p had relatively promising AUCs for BC diagnosis. In the BC cohort, RT-qPCR assay showed that miR-93-5p was significantly elevated in MIBC patients compared with NMIBC and ROC result suggested miR-93-5p exhibited a promising AUC for distinguishing MIBC and NMIBC. Moreover, correlation analysis also showed that urinary exosomal miR-93-5p expression level was significantly related with TNM stage (Ta-T1 vs T2-T4) of BC patients. These results suggested that urinary exosomal miR-93-5p might have the ability to distinguish MIBC and NMIBC. It must be noted that we did not observe a significant difference in the expression level of candidate miRNAs among BC patients with different pathological types. Considering that the sample size was small in our study, more specimens will be needed to clarify whether these urinary exosomal miRNAs have the ability to distinguish different pathological types.

The studies of miR-516a-5p were rare and understanding of miR-516a-5p remained uncertain. In this study, we discovered that miR-516a-5p was overexpressed in bladder cancer tissues and urinary exosomes from BC patients. The urinary exosome-derived miR-516a-5p had the potential to be a non-invasive biomarker for BC. Ye [42] and his colleagues had found miR-516a-5p might act as a tumor suppressor and inhibit the proliferation of non-small cell lung cancer by targeting HIST3H2A gene. More experiments are needed to explore the mechanism of miR-516a-5p in BC.

MiR-93-5p has been found enriched in various types of tumors [43, 44] and mainly played an oncogenic role in the development of malignant neoplasm, including bladder cancer [45]. Sun [43] and et.al had found that miR-93-5p was up-regulated in cervical cancer cells and promotes cancer progression by down-regulating THBS2/MMPS signal pathway. In bladder cancer, Jiang [45] and et.al had indicated that miR-93 plays an oncogenic role by inhibiting expression of PEDF to promote cancer cells proliferation and invasion through, which was partially in accordance with our bioinformatic analysis results. In our bioinformatic analysis, we firstly predicted and selected the candidate target genes of miR-93-5p via mirWalk and TCGA database. GO enrichment and KEGG pathway analysis were performed to explore the possible mechanisms under miR-93-5p and its target genes in bladder cancer. According to the GO/KEGG analysis and a series studies reported before, we assumed that miR-93-5p might act as a tumor promoter in bladder cancer and its target genes were mainly enriched in proteoglycans in cancer, EGFR tyrosine kinase inhibitor resistance, FoxO signaling pathway, PI3K-Akt signaling pathway and MAPK signaling pathway. These pathway might play an important role in the mechanisms under miR-93-5p in development of bladder cancer. PPI network and survival analysis showed that BTG2 was the hub target gene of miR-93-5p and associated with the prognosis of BC patients. Therefore, we conducted a series of in vitro experiment to verified the bioinformatic analysis results. In this study, RT-qPCR validation assay suggested that the expression of miR-93-5p was both significantly up-regulated in BC tissues and human BC cells compared to that in adjacent non-cancerous tissues and normal cells. The CCK-8 and transwell assay showed that overexpression of miR-93-5p promoted the proliferation, migration and invasion of bladder cancer cells. At the same time, we demonstrated that there was an inverse correlation between miR-93-5p and BTG2 expression. Our findings were consistent with those from previous studies and our bioinformatic analysis.

B-cell translocation gene2(BTG2), the first identified gene in BTG/TOB gene family [46], is engaged in many biological processes in tumor development course. The BTG2 is located on band 2, region 3 of the long arm of chromosome 1 and its mRNA translates 158 amino acids. Biological functions of BTG2 mainly includes the following aspects: a) DNA damage repair. Previous studies had demonstrated that DNA damage stimulates p-53 to activated BTG2 expression, which results in down-regulation of cyclinD1 and inhibition of G1/S transition via pRB pathway [47]. b) Anti-migration. BTG2 had been reported to inhibit Src-FAK (focal adhesion kinase) signaling by downregulating reactive oxygen species (ROS) generation and therefore exert a negative effect on cancer cell metastasis [48]. c) Anti-proliferation. Up-regulation of BTG2 activated by p53-dependent or p53-independent NF-κB signaling pathway inhibits cancer cell proliferation [49], and BTG2 also negatively regulates jAK2/STAT3 signaling to exert an anti-proliferation effects on cells [50]. d) Cell death regulation. Ryu et al. [51] found that overexpression of BTG2 induces G2/M arrest and cell death by inhibiting cyclin B1-Cdc2 binding. e) Differentiation regulation. Several studies demonstrated that BTG2 gene participates in the development and differentiation of nerve cells [52]. Farioli-Vecchioli et.al [53] demonstrated that PC3/Tis21 knockout mice display impaired terminal differentiation of hippocampal granule neurons and defective contextual memory. In a word, BTG2 plays important roles in cell differentiation, proliferation, DNA damage repair and cell death.

BTG2 is expressed in different kinds of organs and tissue, such as spleen, lung, prostate, stomach, pancreas and so on [54]. A series of studies demonstrated that BTG2 mainly acted as a tumor suppressor [33, 34, 48] in cancer. Zhang [55] et.al demonstrated that BTG2 is down-regulated in gastric cancer tissue, and overexpression of BTG2 suppresses the proliferation and migration of gastric cancer cells. Wei [36] et.al demonstrated that overexpression of BTG2 inhibits the protein expression of cyclin D1, MMP-1, and MMP-2, and inhibit the growth, proliferation, and invasiveness of the A549 human lung cancer cell line. Several studies demonstrated relationship between BTG2 and miRNAs. Liu [56] et.al discovered that miR-21 enhances the proliferation and suppresses the apoptosis of laryngeal cancer cells via targeting and downregulating BTG2. Xie [7] et.al demonstrated that miR-6875-3p promotes the proliferation, invasion and metastasis of hepatocellular carcinoma by mediating BTG2/FAK/Akt pathway. In this study, we reveal that BTG2 act as s tumor suppressor in bladder cancer and overexpression of miR-93-5p inhibited BTG2 and then promote the proliferation, migration and invasion of bladder cancer cell. But it still needs more experiments to investigate the concrete signaling pathway and mechanisms between miR-93-5p and BTG2 in bladder cancer.

## Conclusion

In summary, we have performed a global and detailed analysis of the urinary exosomal miRNAs profile in BC patients and identified changes in the expression levels of miR-93-5p, miR-516a-5p and miR-940 compared with health controls. Specifically, miR-93-5p, miR-516a-5p and their combinations are demonstrated to be promising biomarkers for diagnosing BC. These results suggest that these urinary exosomal miRNAs might play critical roles in the pathogenesis and development of BC and warrant further study. We also revealed that miR-93-5p promote proliferation, migration and invasion and, and a novel relationship of miR-93-5p down-regulating BTG2 gene expression in BC cells. It is worth to further explore their correlation, molecular mechanism, and therapeutic values deeply. Both of them have clinical significance and should be considered as promising targets for bladder cancer treatment in the future.

## Supplementary Information

**Additional file 1: Supplement Fig. 1.** Heatmap and Venn diagram of differentially expressed UEs-derived miRNA profiles. (A) Heatmap of differentially expresses UEs-derived miRNA (BC vs Healthy Control). (B) Heatmap of differentially expresses UEs-derived miRNA (MIBC vs NMIBC). (C) A Venn diagram showed the up-regulated miRNAs (3 miRNA) between the differential miRNA expression profile from TCGA database and high throughput sequencing technique. (D) A Venn diagram showed there was no down-regulated miRNAs between the differential miRNA expression profile from TCGA database and high throughput sequencing technique.

**Additional file 2: Supplement Fig. 2.** Venn diagram showed the genes shared between predicted target genes of miR-93-5p and down-regulated genes in TCGA datasets.

**Additional file 3: Supplement Fig. 3.** GO enrichment and KEGG pathway analysis of target genes of UE-derived miR-93-5p. The bubble plot(A) and bar plot(B) of target genes GO enrichment analysis of miRNA-93-5p. The bubble plot(C) and bar plot(D) of target genes KEGG pathway analysis of miR-93-5p.

**Additional file 4: Supplement Fig. 4.** PPI network of target genes of miR-93-5p.

**Additional file 5: Supplement Fig. 5.** Expression level of miR-93-5p in bladder cancer tissue and cell lines with or without transfection. (A) The relative expression levels of miR-93-5p detected by RT-qPCR in bladder cancer tissue and corresponding adjacent normal tissue, expression were presented as relative level:log2 (T/N). (B)The relative miR-93-5p levels in bladder cancer cell lines(UM-UC-3 and T24) and non-tumor urothelial cell line SV-HUC-1, detected by RT-qPCR. The miR-93-5p expression level of bladder cancer after transfection. (C) RT-qPCR analysis showed a significant elevation in the expression level of miR-93-5p in bladder cancer cells transfected with miR-93-5p mimic compared with NC. (D) A significant decrease in the expression level of miR-93-5p was detected in bladder cancer cells transfected with miR-93-5p inhibitor.*** represents p<0.001.

## Data Availability

The datasets used and/or analyzed during the current study are available within the manuscript and its supplementary information files. The results shown here are in whole or part based upon data generated by the TCGA Research Network: https://www.cancer.gov/tcga. Accessed 21 Sept 2020.

## Abbreviations

AUC: area under the receiver-operating characteristic curve
BC: Bladder cancer
BTG2: B cell translocation gene 2
CI: confidence interval
FBS: Fetal bovine serum
GO: Gene Ontology
KEGG: Kyoto Encyclopedia of Genes and Genomes
lncRNA: Long non-coding RNA
mRNA: messenger RNA
miRNA: MicroRNA
MIBC: muscle-invasive bladder cancer
NMIBC: muscle-invasive bladder cancer
NTA: Nanoparticle tracking analysis
OS: Overall survival
PPI: Protein-Protein Internet
ROC: receiver-operating curve
RT-qPCR: Quantitative reverse-transcription polymerase chain reaction
TNM: Tumor-node-metastasis
TEM: Transmission electron microscopy
TCGA: The cancer genome atlas
UE: Urinary exosome
