# Supplementary figures and images for: Urinary Exosomal miRNAs as biomarkers of bladder Cancer and experimental verification of mechanism of miR-93-5p in bladder Cancer
Source: BMC Cancer. 2021 Dec 3;21:1293. doi: 10.1186/s12885-021-08926-x (PMC8641206; doi:10.1186/s12885-021-08926-x)

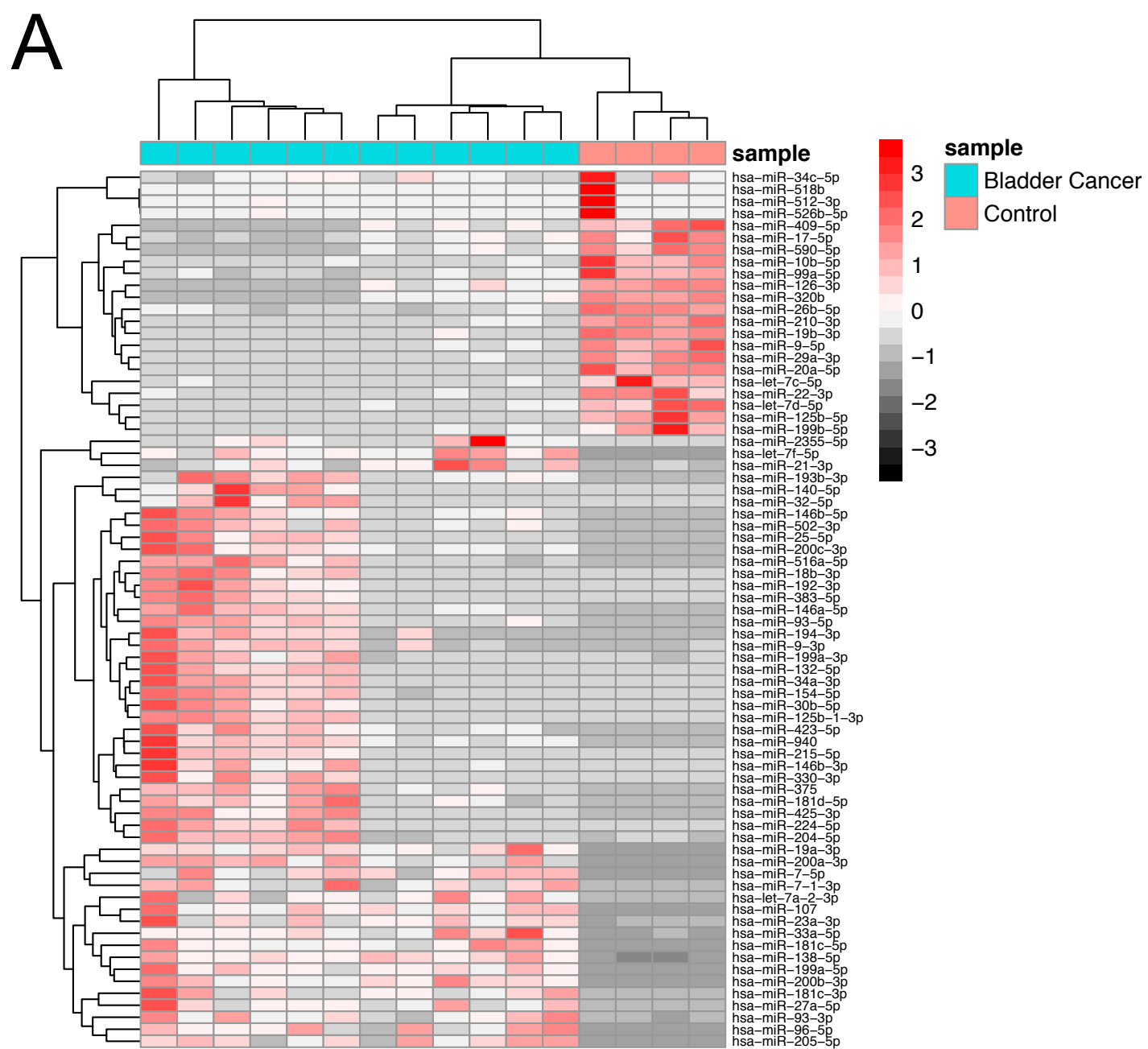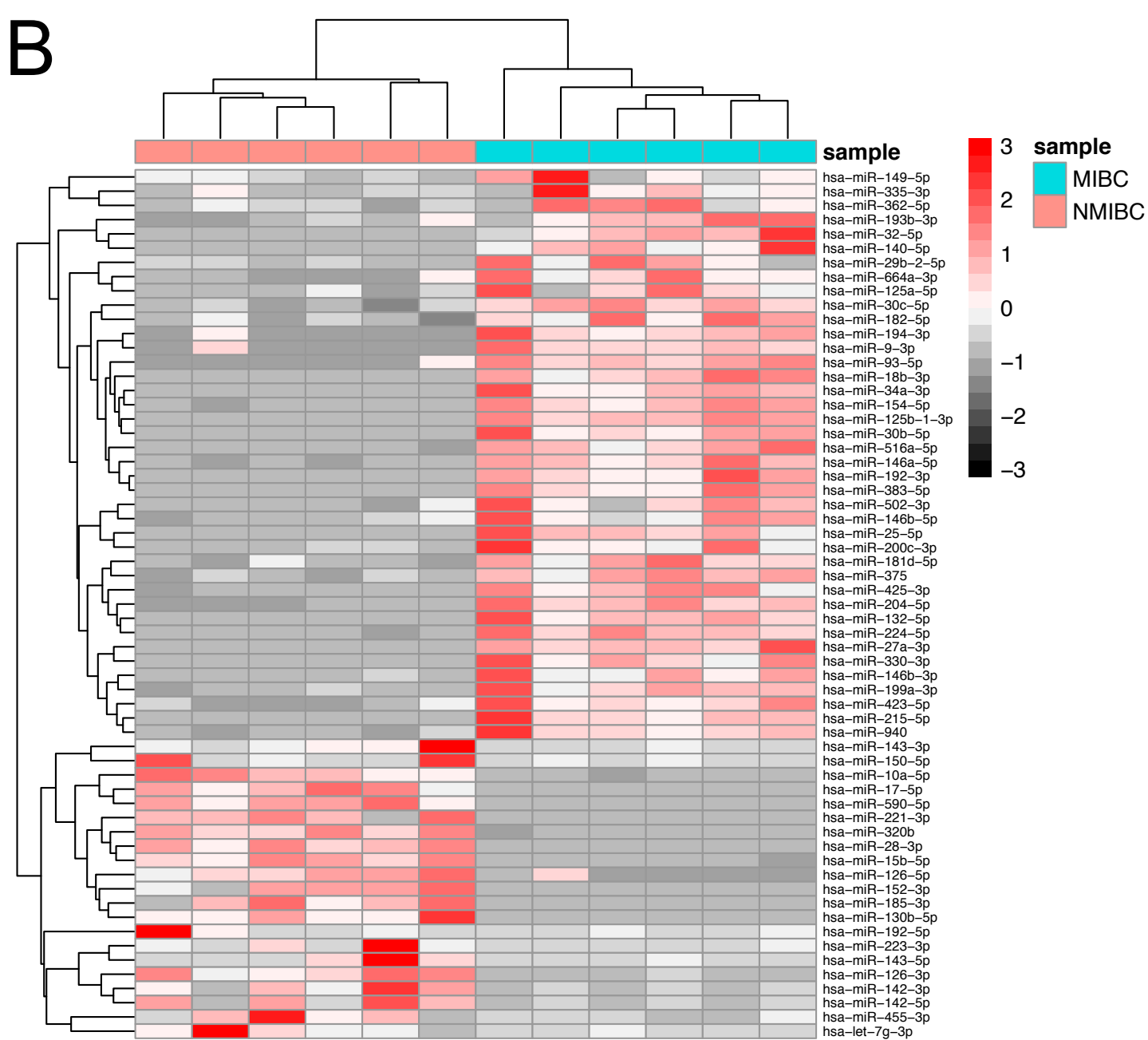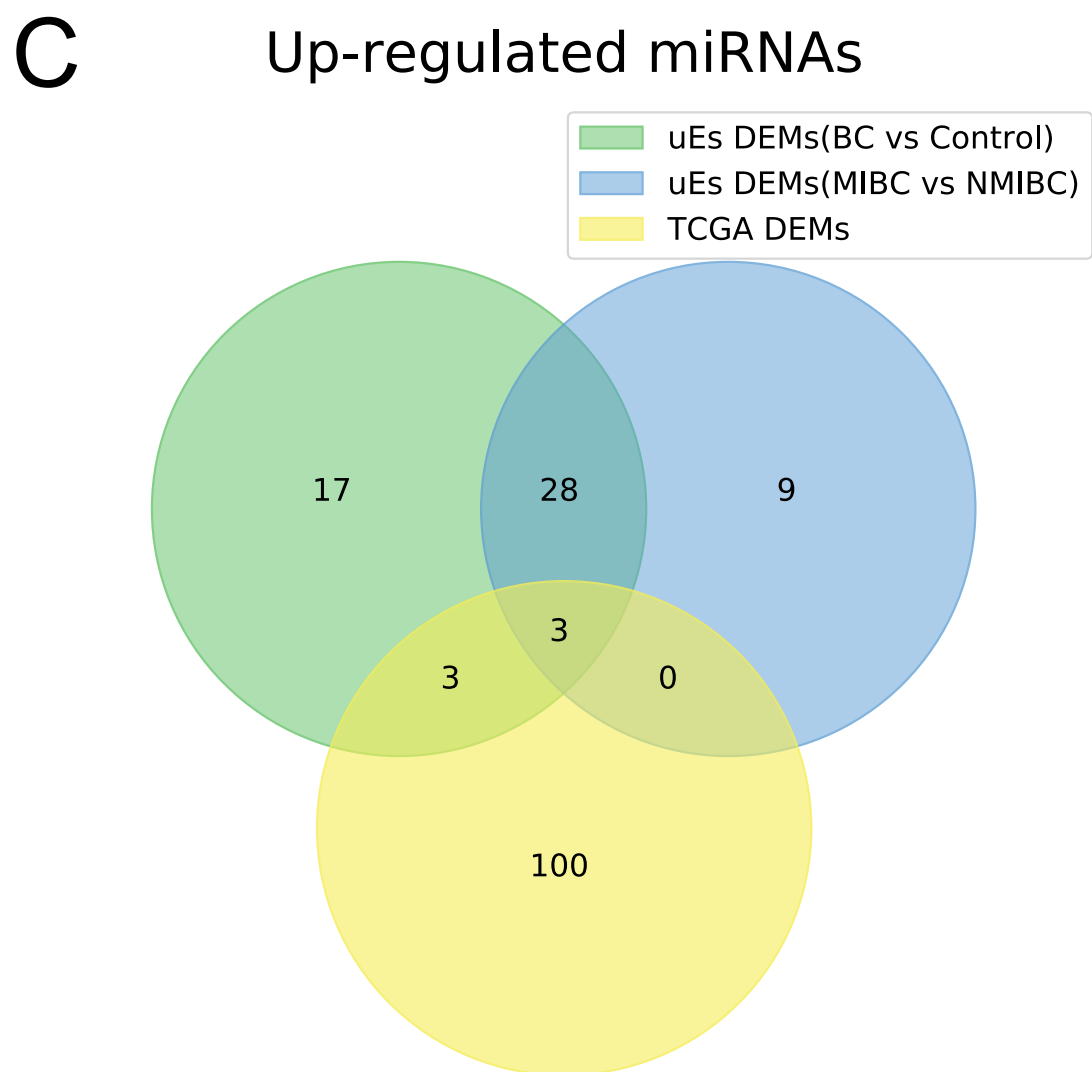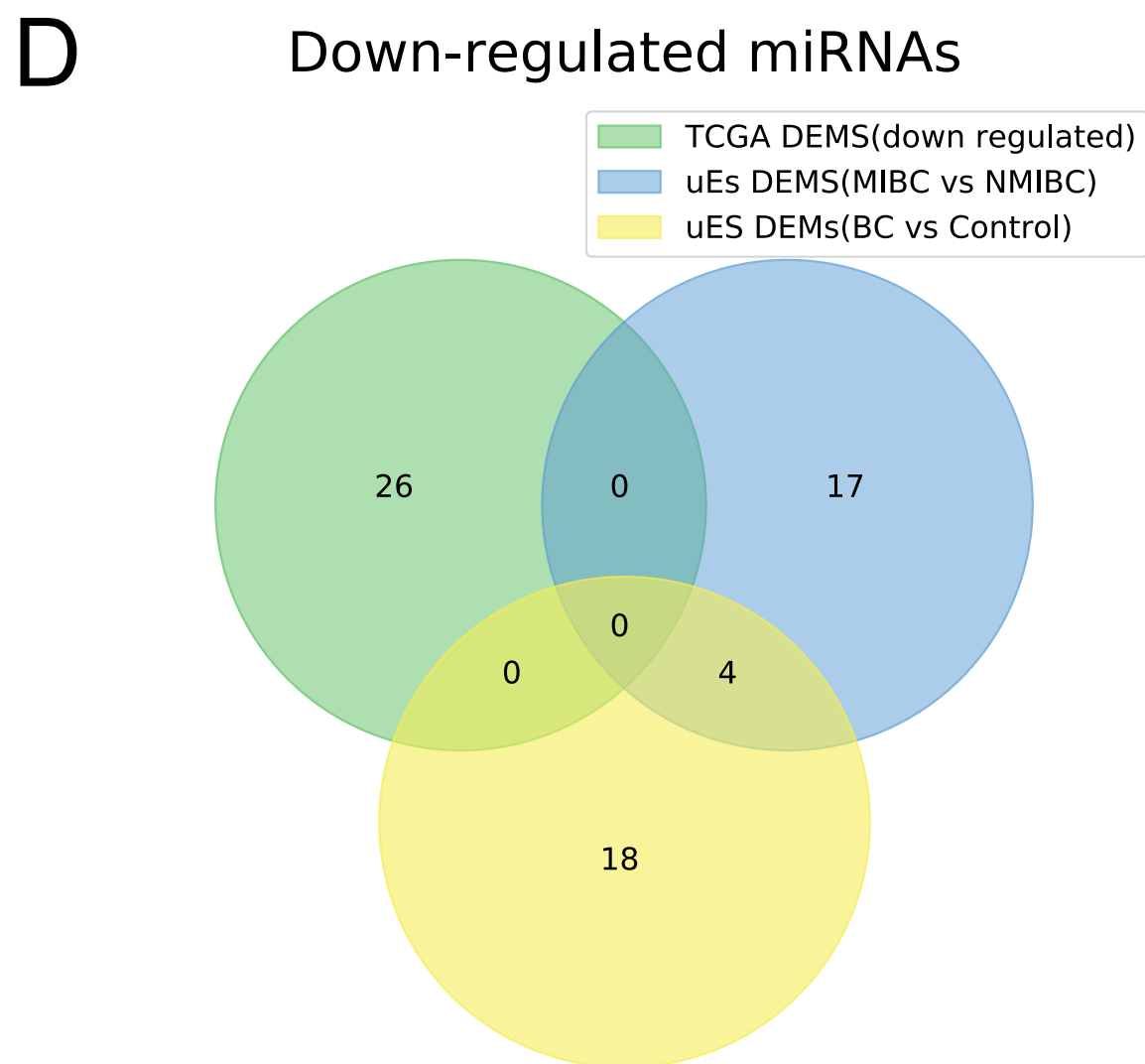

Supplement: Supplementary file 1 — Additional file 1: Supplement Fig. 1. Heatmap and Venn diagram of differentially expressed UEs-derived miRNA profiles. (A) Heatmap of differentially expresses UEs-derived miRNA (BC vs Healthy Control). (B) Heatmap of differentially expresses UEs-derived miRNA (MIBC vs NMIBC). (C) A Venn diagram showed the up-regulated miRNAs (3 miRNA) between the differential miRNA expression profile from TCGA database and high throughput sequencing technique. (D) A Venn diagram showed there was no down-regulated miRNAs between the differential miRNA expression profile from TCGA database and high throughput sequencing technique. [file 12885_2021_8926_MOESM1_ESM.pdf]

# miR-93-5p

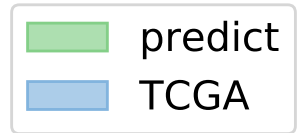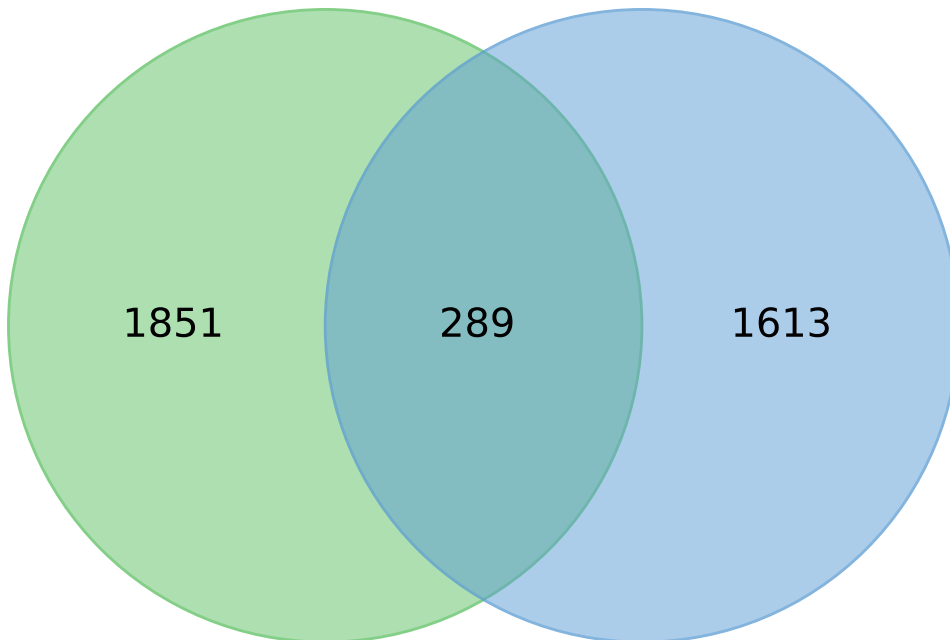

Supplement: Supplementary file 2 — Additional file 2: Supplement Fig. 2. Venn diagram showed the genes shared between predicted target genes of miR-93-5p and down-regulated genes in TCGA datasets. [file 12885_2021_8926_MOESM2_ESM.pdf]

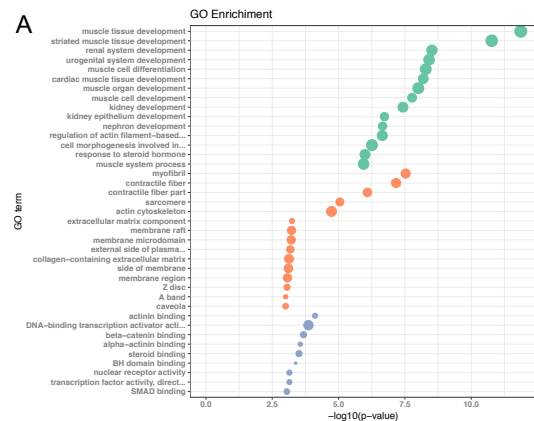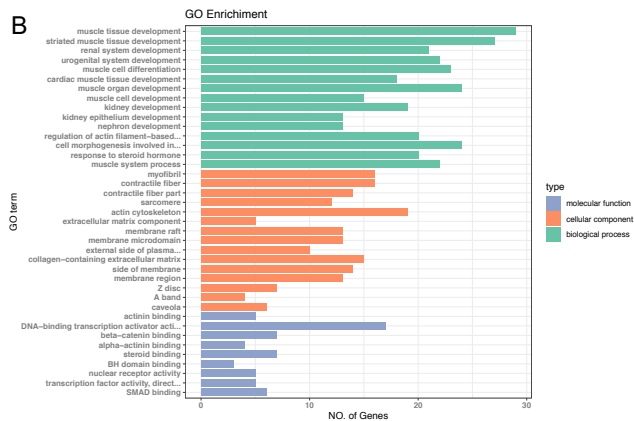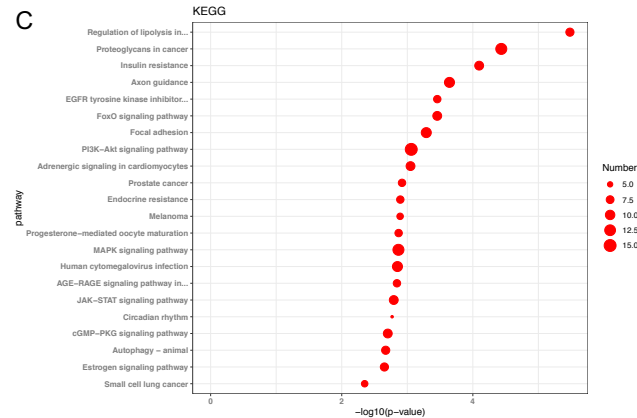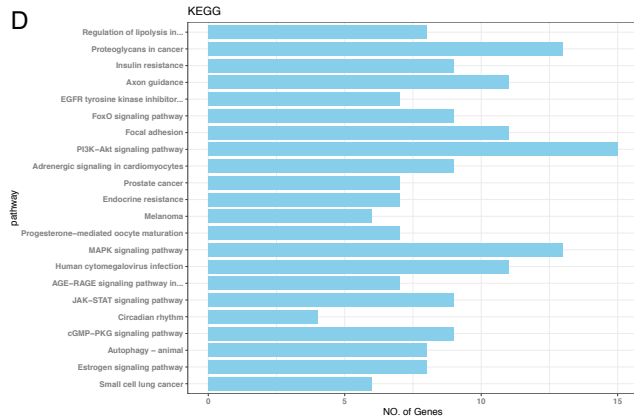

Supplement: Supplementary file 3 — Additional file 3: Supplement Fig. 3. GO enrichment and KEGG pathway analysis of target genes of UE-derived miR-93-5p. The bubble plot(A) and bar plot(B) of target genes GO enrichment analysis of miRNA-93-5p. The bubble plot(C) and bar plot(D) of target genes KEGG pathway analysis of miR-93-5p. [file 12885_2021_8926_MOESM3_ESM.pdf]

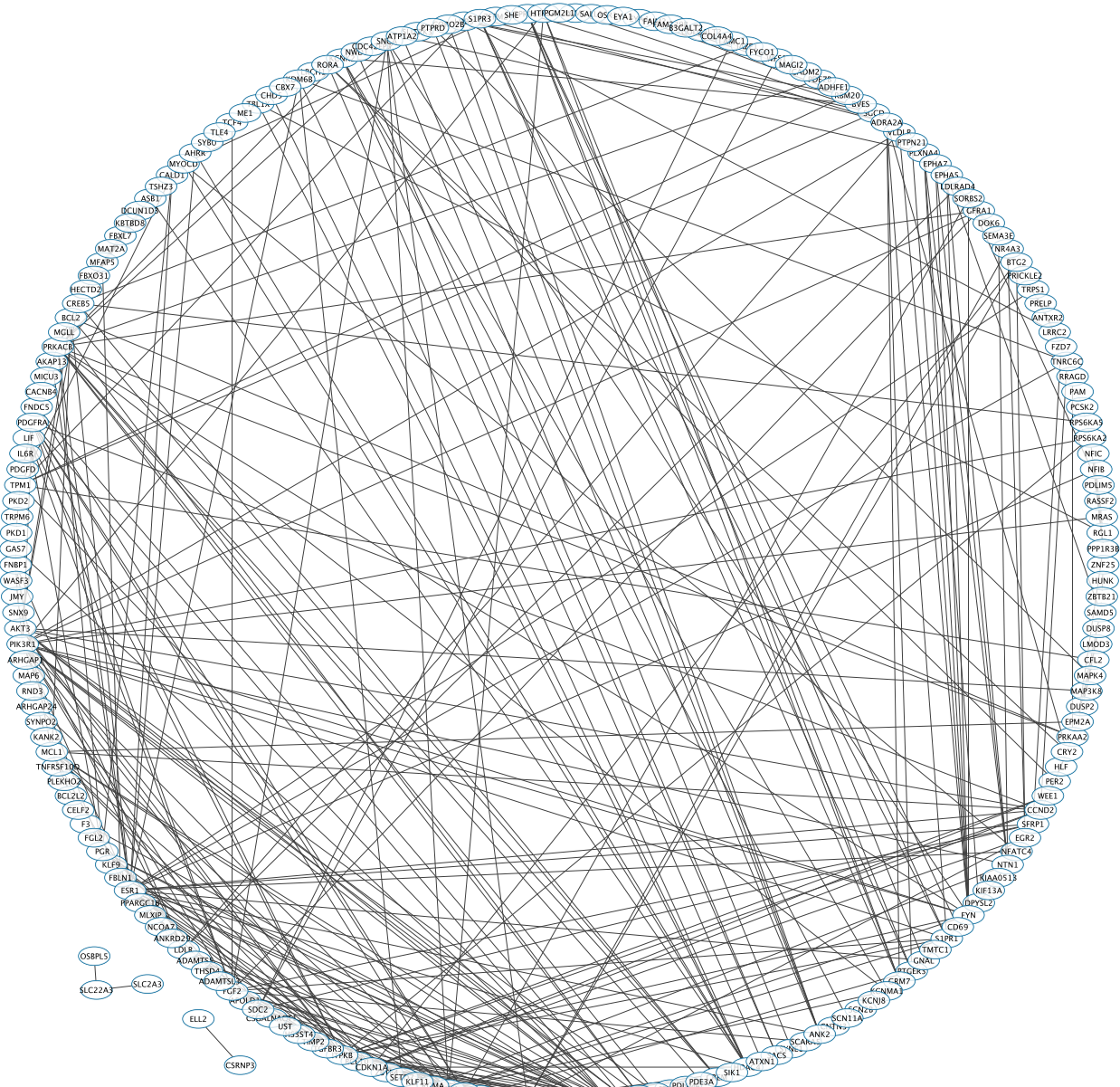

Supplement: Supplementary file 4 — Additional file 4: Supplement Fig. 4. PPI network of target genes of miR-93-5p. [file 12885_2021_8926_MOESM4_ESM.pdf]

**A**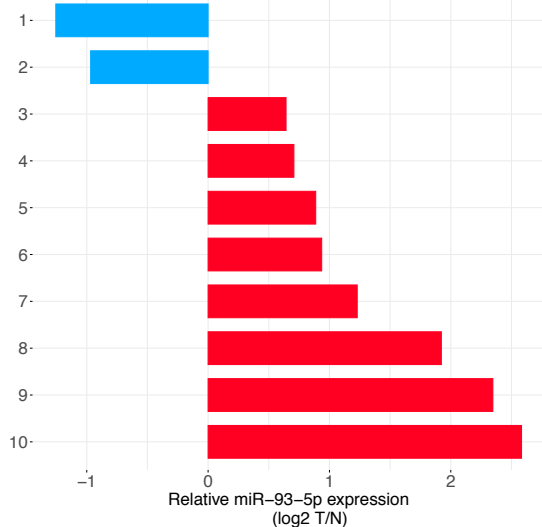**B**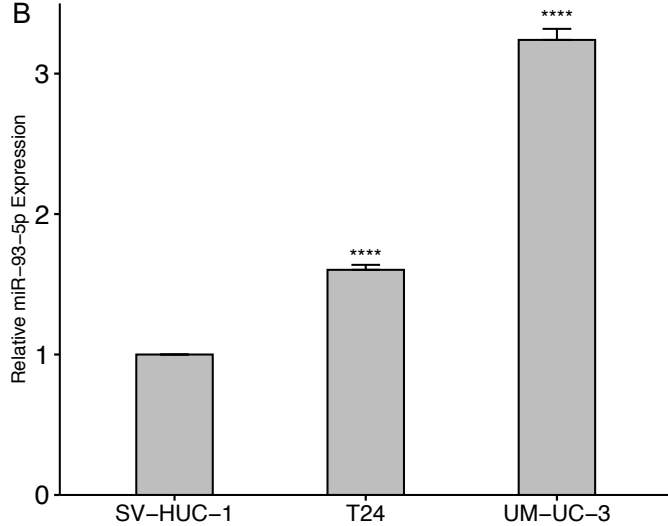**C**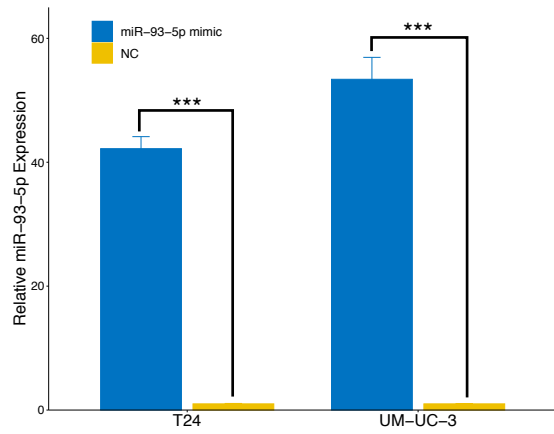**D**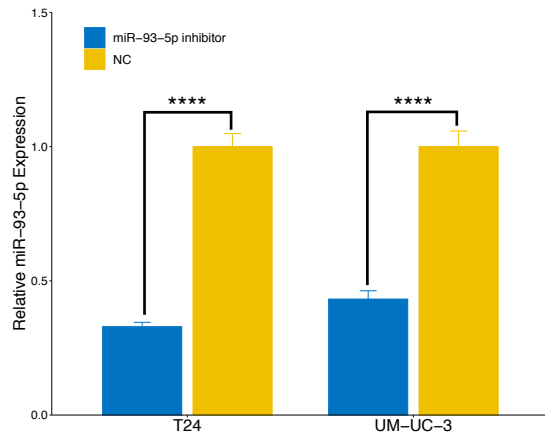

Supplement: Supplementary file 5 — Additional file 5: Supplement Fig. 5. Expression level of miR-93-5p in bladder cancer tissue and cell lines with or without transfection. (A) The relative expression levels of miR-93-5p detected by RT-qPCR in bladder cancer tissue and corresponding adjacent normal tissue, expression were presented as relative level:log2 (T/N). (B)The relative miR-93-5p levels in bladder cancer cell lines(UM-UC-3 and T24) and non-tumor urothelial cell line SV-HUC-1, detected by RT-qPCR. The miR-93-5p expression level of bladder cancer after transfection. (C) RT-qPCR analysis showed a significant elevation in the expression level of miR-93-5p in bladder cancer cells transfected with miR-93-5p mimic compared with NC. (D) A significant decrease in the expression level of miR-93-5p was detected in bladder cancer cells transfected with miR-93-5p inhibitor.*** represents p<0.001. [file 12885_2021_8926_MOESM5_ESM.pdf]
